# Novel S-Bend Resonator Based on a Multi-Mode Waveguide with Mode Discrimination for a Refractive Index Sensor

**DOI:** 10.3390/s19163600

**Published:** 2019-08-19

**Authors:** Do-Hyun Kim, Su-Jin Jeon, Jae-Sang Lee, Seok-Ho Hong, Young-Wan Choi

**Affiliations:** Department of Electrical and Electronics Engineering, Chung-Ang University, 221 Heuksuk-Dong, Dongjak-ku, Seoul 156-756, Korea

**Keywords:** mode discrimination, multi-mode waveguide, S-bend resonator, refractive index sensor, integrated optical sensor

## Abstract

In this paper, a multi-mode waveguide-based optical resonator is proposed for an integrated optical refractive index sensor. Conventional optical resonators have been studied for single-mode waveguide-based resonators to enhance the performance, but mass production is limited owing to the high fabrication costs of nano-scale structures. To overcome this problem, we designed an S-bend resonator based on a micro-scale multi-mode waveguide. In general, multi-mode waveguides cannot be utilized as optical resonators, because of a performance degradation resulting from modal dispersion and an output transmission with multi-peaks. Therefore, we exploited the mode discrimination phenomenon using the bending loss, and the resulting S-bend resonator yielded an output transmission without multi-peaks. This phenomenon is utilized to remove higher-order modes efficiently using the difference in the effective refractive index between the higher-order and fundamental modes. As a result, the resonator achieved a Q-factor and sensitivity of 2.3 × 10^3^ and 52 nm/RIU, respectively, using the variational finite-difference time-domain method. These results show that the multi-mode waveguide-based S-bend resonator with a wide line width can be utilized as a refractive index sensor.

## 1. Introduction

Recently, integrated optical devices for refractive index (RI) sensors have been widely studied for applications such as bio-chemical analysis and temperature monitoring. Structures such as ring resonators, microdisk resonators, Mach—Zehnder interferometers, Fabry—Perot interferometers, and fiber coupler have been developed for use as refractive index sensors [1,2,3,4,5,6,7]. These are based on measurements of the resonance wavelength peak shift through external refractive index changes when a reaction occurs in the sensing region, such as a bio-chemical reaction of the target or a temperature change. Among optical devices, ring resonators such as liquid core optical ring resonators, split ring resonators, and planar ring resonators have been studied in biological and chemical sensing, because these have considerably high Q-factors and steep slopes [8,9,10,11]. Single-mode waveguides have generally been utilized in ring resonators, as they have the advantages of a low propagation loss, small size, and low modal dispersion [12,13]. However, a relatively high-cost fabrication process must be employed, and mass production is difficult as single-mode waveguides typically have widths of several hundred nanometers.

To solve this problem, we proposed and designed a novel S-bend ring resonator, based on a multi-mode waveguide exploiting a mode discrimination phenomenon. Mode discrimination means that high-order modes are removed in specific structures, which yield a performance similar to that of a single-mode waveguide by removing the multi-peaks from the multi-mode waveguide. In a multi-mode waveguide, high-order modes have lower propagation constants than fundamental mode, and so the effective refractive index of a high-order mode is smaller than that of a fundamental mode [14,15]. Because the effective refractive index differs between fundamental and higher-order modes, different bending losses occur on each mode in a bending structure. Therefore, by employing a bending structure yielding a mode discrimination phenomenon based on a multi-mode waveguide, it is possible to solve the problem of the relatively high-cost fabrication process and achieve mass productivity. To exploit this phenomenon, we analyzed and designed an S-bend structure using a multi-mode waveguide to minimize the loss of the fundamental mode and remove higher-order modes. The resonator consisted of the SU-8 2002 polymer, which has a simple fabrication process and good optical transparency [16]. In addition, SU-8 has a smaller refractive index than other materials employed as waveguides, allowing wider line width design of waveguides with the same number of modes [17]. Furthermore, we designed the multi-mode waveguide-based S-bend resonator without multi-peaks, which yields a similar output transmission to a single-mode waveguide-based resonator for MODE simulation by using the variational finite-difference time-domain (varFDTD) method.

## 2. Design of S-Bend Resonator

**Figure 1:**
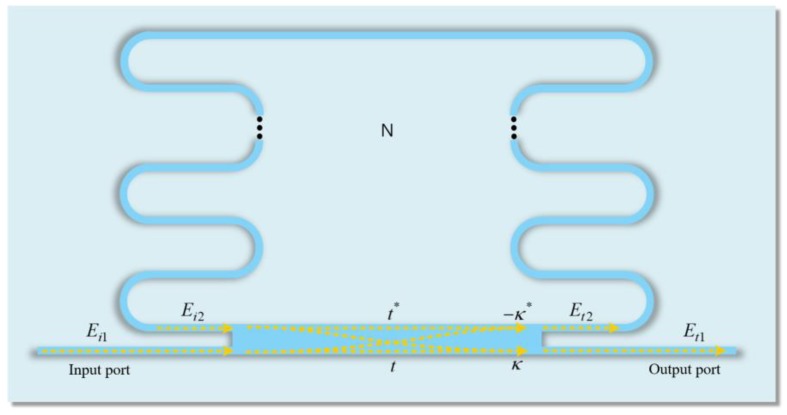
Top-view schematic of the S-bend resonator based on the multi-mode waveguide.

**Figure 2:**
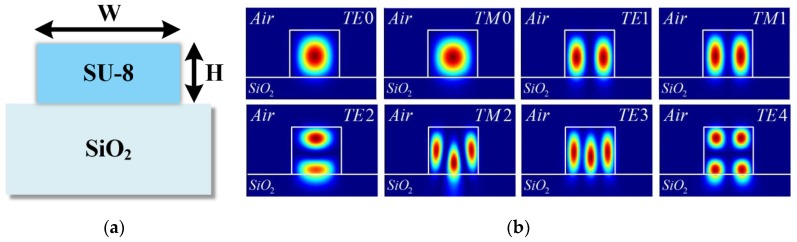
(**a**) Cross-section of the ridge waveguide; (**b**) E-field profiles of SU-8-based waveguide structure.

The S-bend resonator consists of a ridge waveguide, a multi-mode interferometer (MMI) coupler, and several bend structures, as shown in Figure 1. The waveguide is a highly important component of the resonator design, because the line width of the waveguide determines the range and costs of the available fabrication process. To reduce the fabrication process costs, we designed a multi-mode waveguide with a line width of several micrometers. Furthermore, the coupling efficiency between the optical fiber and the waveguide is higher than a single-mode waveguide, because the multi-mode waveguide has a wider line width. As shown in Figure 2a, the multi-mode waveguide was designed on the SiO_2_ ($n_{{\mathrm{SiO}}_{2}}$ = 1.44 at λ = 1.55 μm) substrate. SU-8 2002 polymer ($n_{\mathrm{SU}-8}$ = 1.564 at λ = 1.55 μm) is utilized as the core material to achieve a simple fabrication process and good optical transparency, and the cross section was designed in a rectangle shape with width (W) of 3 μm and height (H) of 2 μm. The propagation loss in the waveguide is negligible because it has very small values compared to the bending loss. In addition, because SU-8 has a smaller refractive index than other materials, a waveguide with the same number of modes can be designed with a wider line width. Figure 2b shows the electric (E) field profiles of eight modes in the designed waveguide.

**Figure 3:**
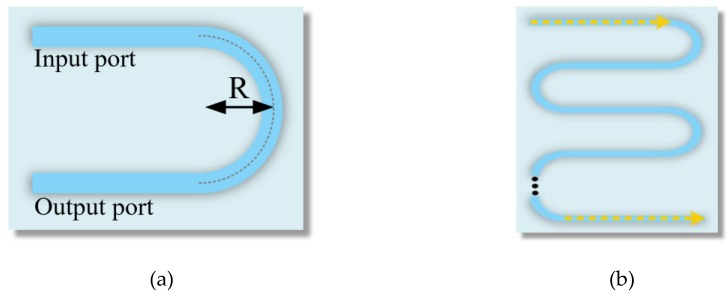
(**a**) Schematic of a semi-circle; (**b**) The S-bend structure for mode discrimination.

However, multi-mode waveguides are not generally suitable for use in resonators owing to a performance degradation and a multi-peak output transmission for higher-order modes. To solve this problem, we exploited the mode discrimination phenomenon that can remove higher-order modes. Furthermore, we analyzed and designed a novel S-bend structure based on the multi-mode waveguide to apply the mode discrimination phenomenon, as shown in Figure 3. The simple approximation of the bending loss is given as follows:

(1) $$\alpha =K\cdot \exp(-cR),\;\mathrm{where}\;c=\beta {\left(\frac{2\Delta n_{eff}}{n_{eff}}\right)}^{3/2}$$

The value of *K* depends on the refractive index of the core and cladding, and also the thickness of the waveguide, Δ*n_eff_* is the difference between the cladding index and modal effective index *n_eff_*, and *R* is the radius of the semi-circle [18]. In Equation (1), it can be observed that the bending loss is inversely proportional to the radius and effective refractive index. The effective refractive index of the mode is expressed by Equation (2).

(2) $$n_{eff}=\frac{\beta }{k_{0}}$$

Here, β is the propagation constant and *k_0_* is the wavenumber in free space. The effective refractive index also decreases as the order of the mode increases in Equation (2), because the propagation constants of high-order modes are smaller than those of the fundamental mode. In other words, the difference in the effective refractive index between the modes leads to different losses in a structure with the mode discrimination phenomenon. Therefore, the multi-mode waveguide can yield a performance similar to that of a single-mode waveguide by removing the higher-order modes. As shown in Figure 3b, we constructed the S-bend structure by cascading the semi-circles shown in Figure 3a.

In general, the bending loss factor in a ring resonator should be reduced [19,20]. However, we focused on the difference in the effective refractive index between the fundamental mode and higher-order modes and analyzed the bending loss according to the radius of the semi-circle for the mode discrimination phenomenon. Higher-order modes with lower effective refractive indexes cause a greater bending loss than does the fundamental mode. Table 1 lists the bending loss (%) for each mode according to the radius of the semi-circle.

**Figure 4:**
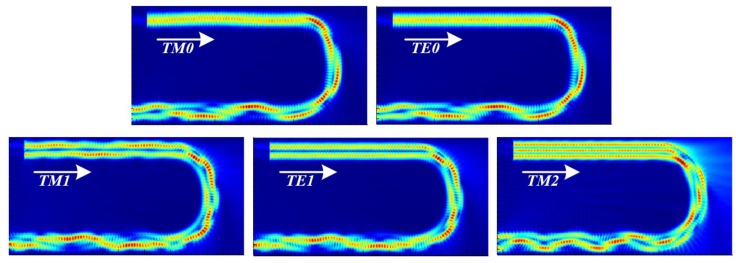
E-field profile of each mode in the semi-circle.

An important factor in our proposed resonator is that there must be some difference in the bending loss between the fundamental and second modes. This is because if the bending loss difference between the fundamental and second modes is very small, then the mode discrimination phenomenon cannot be efficiently applied as the removal ratio of each mode is similar. Furthermore, because the bending loss increases in proportion to the number of semi-circles, the loss of the fundamental mode should be small, and thus a radius of less than 9.5 μm is not suitable. The bending losses at a radius of 10 μm are 2.0% and 2.2% in transverse magnetic (TM) 0 and transverse electric (TE) 0, and 7.8% and 7.9% in TM1 and TE1, respectively. Each difference is approximately 6%. This represents an optimized condition, with a loss difference from the second mode that minimizes the bending loss in the fundamental mode, which is suitable for applying the mode discrimination phenomenon. The higher-order modes above TM2 are omitted from Table 1, as the bending losses tend to be considerably large. Figure 4 depicts the E-field profile for each mode at R = 10 μm. This E-field profile can visually confirm that each mode is removed by the mode discrimination phenomenon in the semi-circle structure.

The S-bend resonator was designed as shown in Figure 1 by applying the previously defined mode discrimination phenomenon at R = 10 μm. The value of N represents the number of layers, each composed of two semi-circles. The resonator was constructed using a multi-mode interferometer (MMI) coupler rather than a direct coupler with a very sensitive ratio change, owing to the nano-scale coupling gap. In addition, the MMI coupler is wider than the designed waveguide, which can allow cost-effective fabrication. The splitting ratio of the MMI is determined by 5:5. This is because the back-reflection of the MMI is minimized at a splitting ratio of 5:5, which improves the performance of the S-bend resonator [17,21].

We can simply analyze the S-bend resonator using the transfer function of a single-ring resonator. Using this model, the relationship in the MMI coupler can be expressed as:

(3) $$\left[\begin{array}{c} E_{t1}\\ E_{t2} \end{array}\right]=\left[\begin{array}{cc} t & \kappa \\ -\kappa^{*} & t^{*} \end{array}\right]\left[\begin{array}{c} E_{i1}\\ E_{i2} \end{array}\right]$$

where *t* and *κ* are the transmission and coupling coefficients, respectively. The round trip in the ring is given by:

(4) $$E_{i2}=\alpha \cdot e^{j\theta }E_{t2}$$

Here, α is the attenuation coefficient considering the propagation and bending losses of S-bend resonator, and *θ* is the phase difference per round trip. Then, the transmitted intensity can be expressed as:

(5) $$P_{t1}={\left|E_{t1}\right|}^{2}=\frac{\alpha^{2}+{\left|t\right|}^{2}-2\alpha \left|t\right|\cos\theta }{1+\alpha^{2}{\left|t\right|}^{2}-2\alpha \left|t\right|\cos\theta }$$

Because the transmitted intensity is affected by the attenuation coefficient, the value of N is critical in designing the resonator. As the value of N increases, mode discrimination can effectively be applied. However, if the value of N becomes too large, then the total loss increases and the performance of the resonator degrades. Therefore, the performance of the resonator according to N is analyzed in Section 3.

## 3. Results and Discussion

**Figure 5:**
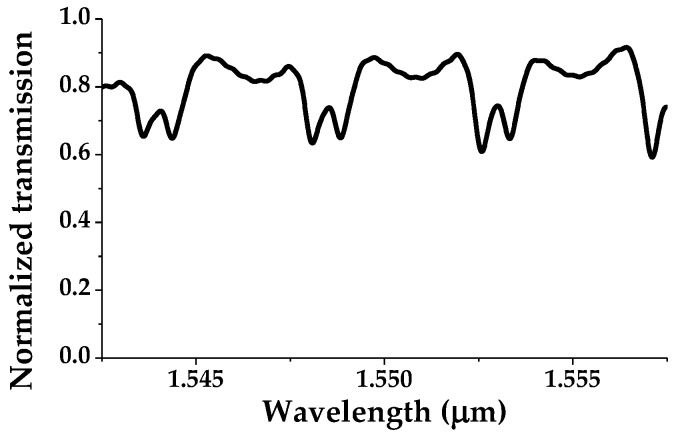
Normalized transmission of S-bend resonator with N = 1.

**Figure 6:**
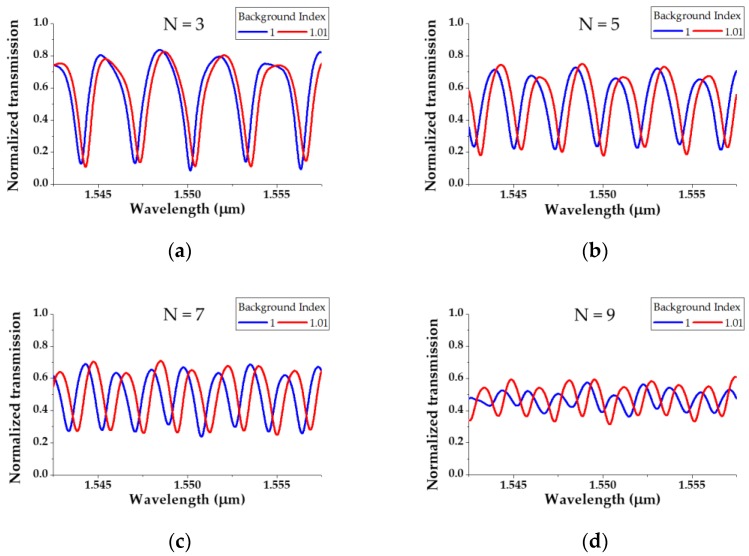
Transmission spectrum according to N when the background index of the S-bend resonator is 1 and 1.01: (**a**) N = 3; (**b**) N = 5; (**c**) N = 7; (**d**) N = 9.

The performance of the resonator was analyzed by fixing the radius at 10 μm and varying N. First, N = 1 represents a stadium type resonator structure composed of two semi-circles. The bending losses of the fundamental and second modes are calculated as (2)^2^ = 4% and (7.8)^2^ = 60.8%, respectively, using the data in Table 1. This indicates that the mode discrimination phenomenon is not properly applied, as higher-order modes of approximately 39% or over remain. Figure 5 shows that the output spectrum exhibits multiple peaks owing to the higher-order modes, making this difficult to employ as an RI sensor. Therefore, we need to remove the higher-order modes more effectively, and so we increased N. Owing to the structural characteristics of the S-bend resonator, only an odd number N can be analyzed. Figure 6 depicts the output transmission of the S-bend resonator according to the background index change (1 to 1.01) when N is 3, 5, 7, and 9. The main performance indicators of the spectra shown in Figure 6 are listed in Table 2. 

The Q-factors for each N are 1.9 × 10^3^, 1.9 × 10^3^, 2.3 × 10^3^, and 2.2 × 10^3^; the sensitivities are 32, 39, 52, and 54 nm/RIU when converted to the refractive index unit (RIU); the limit of detections (LODs) are 2.43 × 10^−4^, 2.1 × 10^−4^, 1.44 × 10^−4^, and 1.23 × 10^−4^ RIU, respectively. As the value of N increases, the higher-order modes are removed more effectively, and so the Q-factor, sensitivity, and LOD increase. However, the decrease in the extinction ratio (ER) as N increases results from a total power loss in the higher-order modes. When N is greater than 9, the ER is 1.36 dB or less, which is too small for usage as an RI sensor. The free spectral ranges (FSRs) are 3.1, 2.24, 1.87, and 1.51 nm when N is 3, 5, 7, and 9, respectively, and the FSR decreases because the length of the resonator increases with N. 

**Figure 7:**
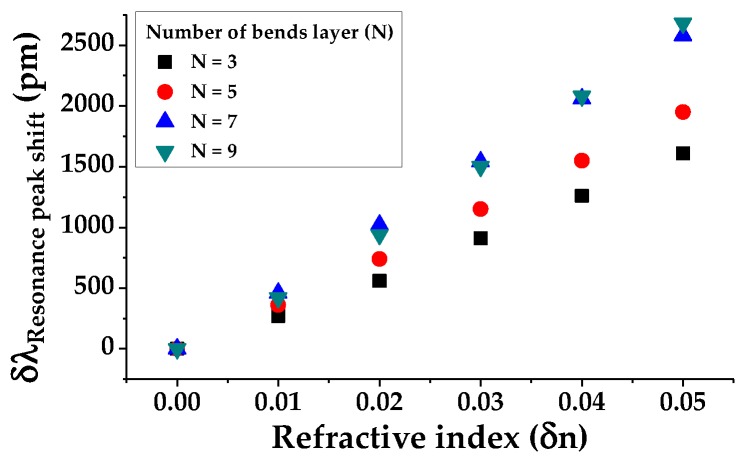
Resonance wavelength peak shift according to N.

**Figure 8:**
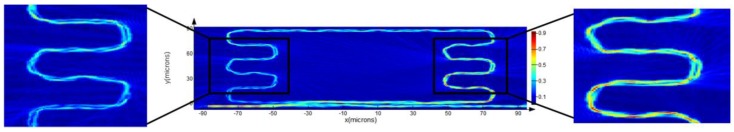
The E-field profile of S-bend resonator with N = 5 at λ = 1.55 μm.

Figure 7 shows the resonance peak shift as the background RI (δn) is varied. As a result of measuring the shifts according to N, when the RI is changed to 0.05 with an interval of 0.01, the sensitivity increases linearly in proportion to N. Figure 8 depicts the E-field profile of the S-bend resonator when N = 5. From the intensities of the E-field on the right and left enlarged profiles, it can be visually observed that the higher-order modes are removed by the bending loss passing through each bend.

## 4. Conclusions

In this paper, we proposed and designed a novel S-bend resonator for an RI sensor based on a multi-mode waveguide exploiting a mode discrimination phenomenon. Conventional multi-mode waveguides suffer from a performance degradation when designing resonators, owing to modal dispersion. To solve this problem, we designed the S-bend structure to exploit the mode discrimination phenomenon. The S-bend resonator using this structure removes the multi-peaks in the output transmission, and yields a similar performance to a single-mode waveguide. Simulation results show that a Q-factor of 2.3 × 10^3^ and sensitivity of 52 nm/RIU can be achieved using the varFDTD method. The sensitivity and Q-factor are slightly lower than those of a single-mode-based ring resonator, but compatible with an RI sensor. Because the S-bend resonator’s line width is greater than those of a single-mode resonator, this can lead to a lower fabrication process cost and higher mass productivity. Thus, the proposed S-bend resonator can be utilized for on-chip refractive index sensors and integrated optical resonator sensors at a competitive price. The mode discrimination phenomenon applied to the S-bend resonator can be utilized in various structures, such as curved structures and total internal reflection mirrors, and will have significant applications in the optical sensor field.

## Figures and Tables

**Table 1:** Bending loss of each mode according to the radius.

	R (μm)	TE0	TM0	TE1	TM1	TM2
Bending loss (%)	12.0	0.8	1.0	3.2	2.8	20.8
	11.5	1.1	1.7	3.3	3.1	48.3
	11.0	1.2	1.8	3.8	3.3	28.6
	10.5	1.5	1.6	5.0	5.2	32.7
	10.0	2.2	2.0	7.9	7.8	46.8
	9.5	3.6	4.0	13.1	14.1	46.2

**Table 2:** Detailed performance indexes of the S-bend resonator according to N.

N	3	5	7	9
**FSR (*nm*)**	3.1	2.24	1.87	1.51
**Q-factor**	1.9×10^3^	1.9×10^3^	2.3×10^3^	2.2×10^3^
**Sensitivity**	32 nm/RIU	39 nm/RIU	52 nm/RIU	54 nm/RIU
**Extinction ratio**	9.7 dB	4.7 dB	4.3 dB	1.36 dB
**Limit of detection**	2.43×10^−4^ RIU	2.1×10^−4^ RIU	1.44×10^−4^ RIU	1.23×10^−4^ RIU
